# Untargeted LC-IMS-qToF-MS-Based Lipidomics Approach to Evaluate the Effect of a Polyphenol-Rich Beverage on Human Lipid Profiles

**DOI:** 10.3390/ijms26199417

**Published:** 2025-09-26

**Authors:** Simone Stegmüller, Marleen Huber, Celina Rahn, Tamara Bakuradze, Jens Galan, Elke Richling

**Affiliations:** 1 Division of Food Chemistry and Toxicology, Department of Chemistry, RPTU University Kaiserslautern-Landau, Erwin-Schrödinger-Straße 52, 67663 Kaiserslautern, Germany; simone.stegmueller@chem.rptu.de (S.S.); huber@rhrk.uni-kl.de (M.H.); goettelc@rhrk.uni-kl.de (C.R.); bakurad@rhrk.uni-kl.de (T.B.); 2 Medical Institute, Hochgewanne 19, 67269 Grünstadt, Germany; mail@galan.info

**Keywords:** untargeted lipidomics, polyphenols, red fruit, juice, human plasma samples

## Abstract

Polyphenols are an important class of secondary metabolites that possess antioxidant or anti-inflammatory properties and are associated with many health benefits. It has been reported that extracts of fruit juices or the fruit juices themselves are able to influence lipid metabolism. The aims of this study were to establish a reliable analytical method and thereafter investigate the influence of a polyphenol-rich fruit juice during an eight-week intervention on plasma lipid profiles in healthy male subjects. A placebo-controlled intervention study with 36 healthy male subjects was carried out. Volunteers consumed 750 mL of a polyphenol-rich or placebo beverage on a daily basis. With the established untargeted LC-IMS-qTof method, lipids could be identified, and changes in the lipidome could be detected. For the first time, a comparison of the lipidome of the control vs. treatment group allowed for the identification of differences in lipid profiles. The observed changes suggest that polyphenol intake leads to the targeted re-modeling of the lipidome, affecting bioactive lipid mediators and membrane components in particular. In the future, our identified lipid markers may be established as potential biomarker candidates related to health.

## 1. Introduction

The regular consumption of fruits, vegetables, and other foods rich in antioxidants contributes to general health and is associated with lower incidences of cardiovascular and other chronic diseases [1,2,3]. They are the most ubiquitous sources of phenolic compounds, mainly flavonoids and hydrolysable tannins, which mostly contribute to their antioxidative activity. Polyphenols exhibit a strong protective effect against cellular oxidative damage. Recent studies have revealed an important role of polyphenol-rich fruit juices, such as pomegranate, cranberry, and chokeberry juices, in protecting biological membranes by decreasing lipid peroxidation in both humans and atherosclerotic mice [2,4,5,6,7]. In addition to these antioxidant and anti-inflammatory properties, polyphenol-rich products exert DNA protective effects, and they can decrease blood pressure, triglycerides (TGs), and total and low-density lipoprotein (LDL) cholesterol levels and increase high-density lipoprotein (HDL) cholesterol concentrations in humans [2,5,7,8]. Thus, polyphenol-rich fruit juices seem to have a great influence on body composition and, in particular, on lipid changes. However, their effects on other lipid class compositions in humans, such as polar lipids, have scarcely been investigated.

Polar lipids, more specifically phospholipids (PLs) and sphingolipids (SLs), are important biomolecules that not only constitute the structural building blocks and scaffolds of cell and organelle membranes but also play an active role in cell biochemistry, physiology, and pharmaceuticals [9]. Indeed, they are involved in signal transmission and cell recognition, thus playing an essential role in the regulation of cell growth, differentiation, senescence, and apoptosis [10]. PLs, SLs, and the products of their enzymatic metabolism may have widespread effects on pathways related to inflammation; lipid metabolism, such as cholesterol metabolism; and HDL function [11,12,13]. As a consequence of the multiple functions of PLs and SLs, alterations in their metabolism are associated with several diseases, such as cancer, immune dysfunction, diabetes, liver disease, and inflammation [14,15,16,17]. Considering all these facts, the identification and quantification of PLs and SLs are of high interest in lipidomics for understanding their biological roles and mechanisms in specific diets or certain diseases. Further, they would allow for the identification and validation of biomarkers or even the characterization of the beneficial effects of food on lipid profiles.

The main difficulty in the exploration of the phospholipidome and sphingolipidome lies in the great diversity of their structures. This complexity is reflected not only by the nature of the hydrophilic head that defines the subclass of PLs and SLs but also by the hydrocarbon backbones composed of one or two aliphatic chains at distinct positions (i.e., sn1 versus sn2). The latter can also contain several double bonds, whose position and stereochemistry further increase the diversity of these lipids.

Thus, untargeted lipidomics represents a real analytical challenge, and the identification of compounds certainly remains a key factor. In addition, the results obtained from these data are complex, as one sample corresponds to one total ion current, and each retention time unit is associated with a full-scan mass spectrum. Thus, sophisticated algorithms are needed to ensure robust data processing and allow, among other things, retention time alignment, deisotoping, and feature extraction [18].

This study aimed to establish a reliable analytical method for an untargeted lipidomics approach and thereafter investigate whether a polyphenol-rich beverage modifies plasma lipid profiles after 8 weeks of consumption in healthy male subjects.

## 2. Results

### 2.1. Lipid Coverage in Plasma Samples

Following the validation of the method, plasma samples obtained from a human intervention study were measured. In addition to the samples obtained from the study participants, quality control (QC) samples were prepared from an independent blood sample taken from a female individual. These QC samples were measured at regular intervals between the study samples to assess the performance of the method over the measurement period.

**Figure 1:**
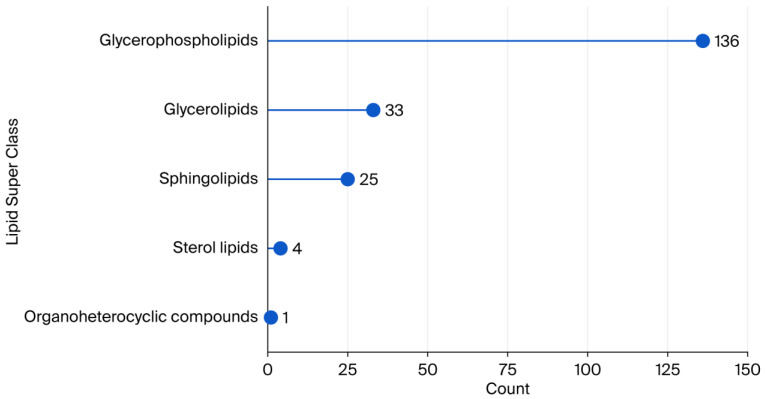
Counts of lipid super classes annotated in human blood plasma samples with the untargeted lipidomics approach.

After data processing and annotation, the coverage of the identified lipids was examined. The application of this untargeted lipidomics approach resulted in the identification of 199 lipids, predominantly glycerophospholipids, in the plasma samples (see Figure 1). A comprehensive review of the extant literature revealed that nearly 600 structural forms of lipid molecules have been reported in human plasma samples [19].

**Figure 2:**
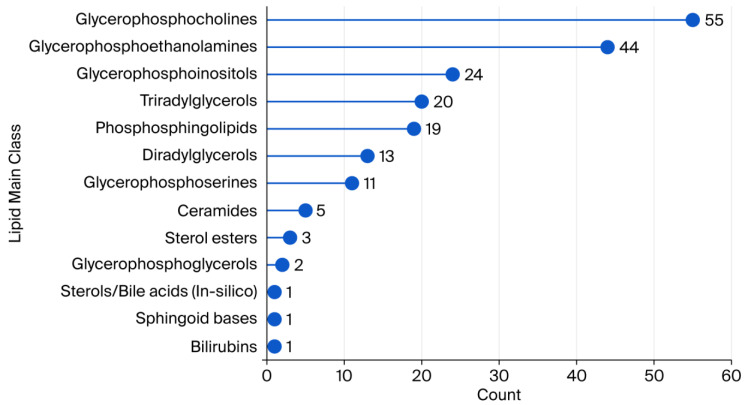
Counts of lipid main classes annotated in human plasma samples with the untargeted lipidomics approach.

As illustrated in Figure 2, a wide array of primary lipid categories could be identified. The data demonstrated that glycerophosphocholines (54 compounds) and glycerophosphoethanolamines (44 compounds) were the most prevalent classes, followed by glycerophosphoinositols (24), triacylglycerols (TAGs) (20), and phosphosphingolipids (19). A range of 11 to 5 identified compounds was observed in other lipid categories, including diradylglycerols, glycerophosphoserines, and ceramides. It was determined that minor classes, such as bilirubins, sphingoid bases, and sterols/bile acids, comprised only four identified molecules each. These results show a heterogeneous distribution of lipid species, emphasizing the dominance of glycerophospholipids in the measured lipid profiles.

**Figure 3:**
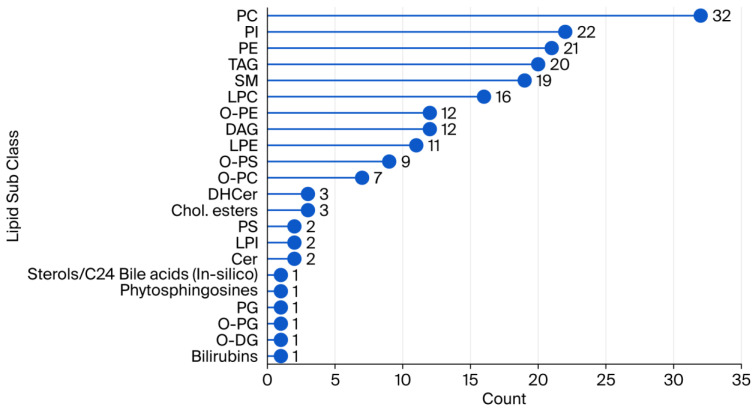
Counts of lipid subclasses annotated in human plasma samples with the untargeted lipidomics approach.

The most abundant subclass (Figure 3) was phosphatidylcholines (PCs), with 32 identified compounds, followed by phosphatidylinositols (PIs) (24), phosphatidylethanolamines (PEs) (22), triacylglycerols (TAGs) (21), and sphingomyelins (SMs) (20). It is noteworthy that several other subclasses, including lysophosphatidylcholines (LPCs), ether-linked phosphatidylethanolamines (O-PEs), and diacylglycerols (DAGs), exhibited a range of 12 to 16 identified compounds. Lower-abundance subclasses, such as bilirubins, ether-linked di-acylglycerols (O-DGs), and phosphatidylglycerols (PGs), contained only 1–2 compounds. This distribution is indicative of the chemical diversity of the lipidome. The most prevalent molecular species identified were phospholipids and neutral lipids.

### 2.2. Comparison of the Intervention Groups and Blood Sampling Times

As part of an intervention study performed by our group [20], blood samples were taken from 36 study participants at three different times after overnight fasting (blood sampling dates (BSDs)); additionally, as described in Section 4.3, plasma was collected, and lipids were extracted. On the first BSD, all subjects had completed a one-week washout phase, during which they had to follow a low-polyphenol diet. This was adhered to by all participants throughout the entire eight-week study period. After the one-week washout phase, the participants were separated into two test groups (a parallel study design) receiving two different test drinks. The intervention group received 750 mL of a polyphenol-rich beverage based on red fruits (chokeberry, cranberry, and pomegranate) daily, while the control group received 750 mL of an isocaloric test beverage that differed only in its sorbitol content (8.7 g/L vs. 0 g/L) and polyphenol content (3.0 g gallic acid equivalents (GAE)/L vs. 0.1 g GAE/L) (for a detailed composition of the test beverage and polyphenol profile, see [20]). The second blood sample (BSD 2) was collected after a 4-week period of consumption of the test beverages, and the third blood sample was obtained at the end of the 8-week study.

**Figure 4:**
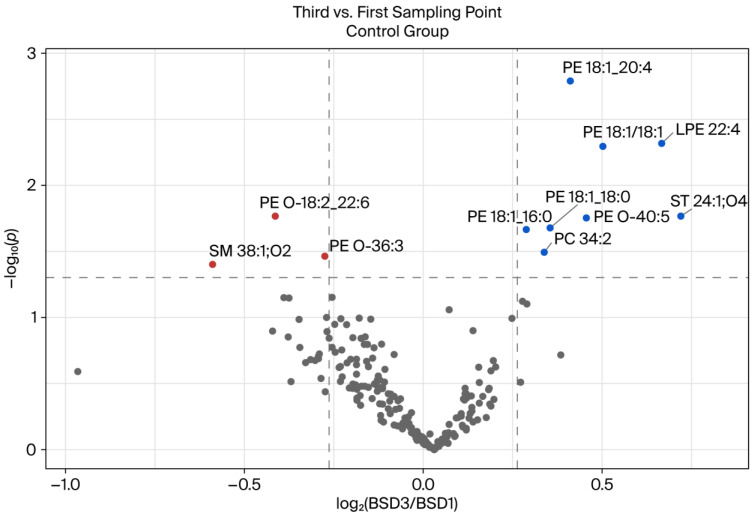
Volcano plot showing the differences between blood sampling dates (BSDs) 1 and 3 of the control group. The fold change is set to 1.2, and the significance level is *p* < 0.05. The lipids that changed significantly in the comparison of the two groups are shown in red (decreased) and blue (increased). All annotated lipids are included. The samples were prepared in duplicate and measured in *n* = 2.

The volcano plot illustrated in Figure 4 provides a visualization of the disparities between the initial and third blood sampling dates (BSDs) within the control group. This analysis revealed the presence of eleven lipids that exhibited a statistically significant difference (*p* > 0.05) with a minimum 1.2-fold variation compared to the initial BSD. These can be observed in the volcano plot as red (decreased) and blue (increased) circles. In the control group, after the eight-week intervention of consuming the placebo drink (without polyphenols), eight lipids were found to have increased significantly by at least 1.2-fold, namely, PC 34:2, ST 24:1;O4, LPE 22:4, PE O-40:5, PE 18:1:20:4; PE 18:1_16:0, PE 18:1/18:1, and PE 18:1_18:0. Furthermore, a significant decrease in the levels of the three lipids SM 38:1;O2, PE O-18:2_22:5, and PE O-36:3 was observed between the two blood sampling dates in this group. The most significant change was observed in the lipid ST 24:1;O4. Moreover, the lipids that demonstrated a decrease following the eight-week intervention exhibited a diminished level of significance. Furthermore, an analysis of the PE subclass revealed that it contained the largest proportion of lipids that had undergone significant changes, with eight lipids exhibiting alterations, including the lyso compound. 

**Figure 5:**
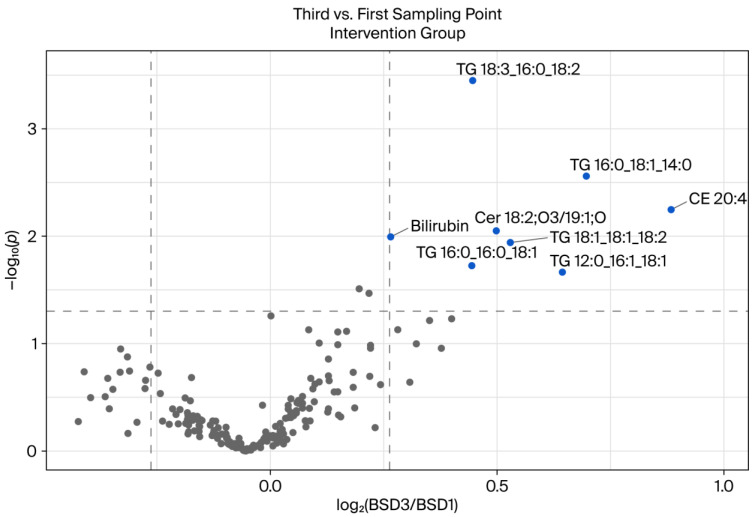
Volcano plot showing the differences between blood collection dates 1 and 3 of the intervention group. The fold change is set to 1.2, and the significance level is *p* < 0.05. The lipids that changed significantly in the comparison of the two groups are shown in blue (increased). All annotated lipids are included. The samples were prepared in duplicate and measured in *n* = 2.

Between the first and third blood sampling dates in the intervention group (see Figure 5), a total of eight lipids demonstrated a significant change of at least 1.2-fold compared to the previous blood sampling date. Notably, all of the altered lipids increased in comparison. These lipids included Cer 18:2;O3/19:1;O, TG 12:0_16:1_18:1, TG 18:3_16:0_18:2, TG 16:0_18:1_14:0, TG 18:1_18:1_18:2, TG 16:0_16:0_18:1, and CE 20:4. Bilirubin levels were also measured, and they exhibited a substantial increase. A notable observation is that, among the eight lipids that exhibited a significant alteration, a total of five lipids belonging to the lipid class glycerolipids and the category of triglycerides or triacylglycerides were identified. The triglyceride TG 18:3_16:0_18:2 demonstrated the most substantial change. Notably, the most substantial change was observed in the lipid CE 20:4. Bilirubin also differed significantly, but the fold change limit was only just about exceeded. Given that bilirubin is not classified as a lipid, it is assigned a subordinate role in the context of lipidomics. 

**Figure 6:**
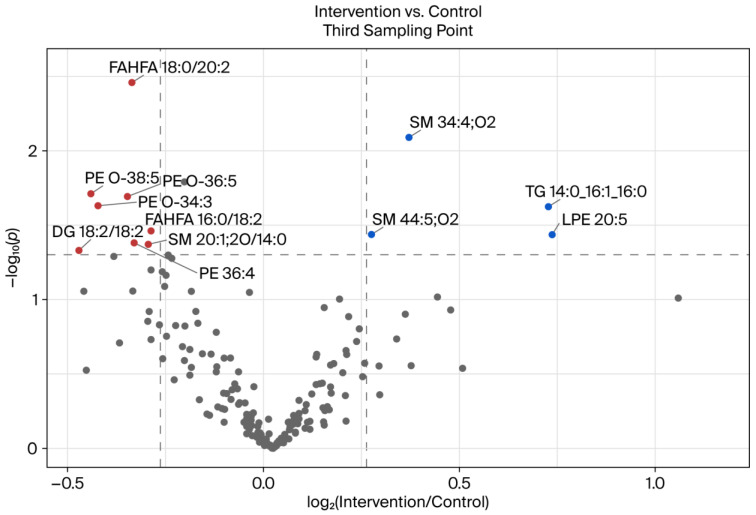
Volcano plot showing the differences between the intervention and control groups at the third blood sampling date. The fold change is set to 1.2, and the significance level is *p* < 0.05. The lipids that changed significantly in the comparison of the two groups are shown in red (decreased) and blue (increased). All annotated lipids are included. The samples were prepared in duplicate and measured in *n* = 2.

The volcano plot in Figure 6 illustrates the significant differences in lipids between the study groups on the third BSD. It is evident that a total of twelve lipids underwent substantial changes or exhibited differences between the study groups. The four lipids SM 34:4;O2, SM 44:5;O2, TG 14:0_16:1_16:0, and LPE 20:5 exhibited a significant increase in the intergroup comparison. Conversely, a substantial decline was evident in the remaining eight lipids: PE O-34:3, SM 20:1;2O/14:0, PE 36:4, PE O-36:5, DG 18:2/18:2, PE O-38:5, FAHFA 16:0/18:2, and FAHFA 18:0/20:2. The two lipids PE O-36:5 and PE O-38:5 were excluded from further analysis due to their significant differences between the study groups in the initial blood sampling. The most significant difference between the two study groups was observed in the lipid LPE 20:5, the levels of which increased by approximately 1.6-fold between the two groups at the study’s endpoint following eight weeks of intervention. A general observation indicated that lipids of the glycerophospholipid class, including PE and LPE, demonstrated a significant discrepancy between the control and intervention groups during the third BE. However, a contrasting trend was observed in the non-lyso compounds PE O-34:3 and PE 36:4, which exhibited a decrease in comparison to the lyso compound. It is noteworthy that two lipids of the fatty acyl class (FAHFA 16:0/18:2 and FAHFA 18:0/20:2) also exhibited a decline. In addition, two lipids belonging to the sphingomyelin (SM) category (SM 34:4;O2 and SM 44:5;O2) were found to be induced. Furthermore, an analysis of the induced quantity or intensity of the triacylglyceride TG 14:0_16:1_16:0 revealed its presence.

Overall, substantial variations in lipid profiles were observed both within the test groups over the 8-week intervention period and between the two groups at the end of the study.

## 3. Discussion

### 3.1. Comparison of BSDs 1 and 3 of the Control Group

We evaluated the changes in the plasma lipidome over time in the control group by comparing the lipid profiles at the first (after a one-week washout period) and third (after eight weeks) sampling time points. Although the participants did not receive any pharmacological treatment or nutritional intervention, they were instructed to adhere to a strict polyphenol-free diet, whereby excessive fat consumption was also prohibited throughout the study period. Figure 4 shows that most lipid species remained stable over time. However, a subset of lipids exhibited statistically significant changes, indicating temporal variations in lipid metabolism, even under tightly controlled dietary conditions. Several PE species increased over time, including PE 18:1_20:4, PE 18:1/18:1, and PE 18:1_18:0, as well as LPE (LPE 22:4) and sulfatide (ST 24:1;O4). These lipids are involved in membrane remodeling, signaling, and immune modulation [21,22]. An increase in these lipids may represent subtle metabolic adaptations to environmental or physiological conditions that accumulate over time, even in the absence of external interventions.

Interestingly, ether-linked phospholipids (e.g., PE O-18:2_22:6 and PE O-36:3) and SMs (e.g., SM 38:1 O2) exhibited notable reductions. Ether lipids are known to contribute to protection against oxidative stress and maintain membrane integrity [23]. It was hypothesized that the observed reduction may be associated with the absence of dietary polyphenols, which are recognized for their antioxidant capacity and ability to modulate lipid metabolism [24]. In the absence of polyphenols in the diet, the body may undergo compensatory adjustments in lipid pathways associated with oxidative defense.

The controlled polyphenol-free diet offered unique insights into how the lipidome responds to the absence of specific dietary components. Polyphenols have been demonstrated to exert an influence on plasma lipid profiles by modulating lipogenesis, lipid oxidation, and inflammation [25]. Their removal likely induced a distinct metabolic state that, while consistent across participants, still permitted quantifiable intra-individual changes over time.

These findings underscore the dynamic nature of lipid metabolism, even under relatively stable dietary conditions. The presence of significant longitudinal changes in the control group emphasizes the necessity of incorporating well-characterized baseline data and time-matched controls in lipidomics studies. Furthermore, it is posited that even controlled diets can influence the lipidome in ways that may be relevant for interpreting the effects of experimental interventions.

### 3.2. Comparison of BSDs 1 and 3 of the Intervention Group

In the intervention group, which received a daily intake of 750 mL of a polyphenol-rich beverage, in addition to adhering to a polyphenol-free background diet, significant changes in the plasma lipidome were observed between the first and third sampling time points. The volcano plot (Figure 5) revealed a distinct metabolic response compared to the control group (Figure 4), with multiple lipid species demonstrating statistically significant increases over time. Several TG species, including TG 18:3_16:0_18:2, TG 16:0_18:1_14:0, TG 16:0_16:0_18:1, and TG 12:0_16:1_18:1, were found to be elevated. Triglycerides play a pivotal role in energy storage and lipid transport. Modulation of these molecules can be indicative of alterations in lipid absorption, hepatic lipid handling, or peripheral lipid turnover. Polyphenols have been demonstrated to impact triglyceride metabolism via several mechanisms, including the activation of AMP-activated protein kinase (AMPK) and the inhibition of de novo lipogenesis [26,27]. The increase in specific TG species may be indicative of enhanced hepatic export of triglycerides or altered fatty acid remodeling in response to polyphenol exposure.

Furthermore, increases in the levels of cholesteryl ester CE 20:4 and certain ceramide species, such as Cer 18:2;O3/19:1;O, were observed. Elevated CE levels may be indicative of altered cholesterol esterification or the mobilization of fatty acid pools associated with inflammation and oxidative balance. Ceramides are bioactive sphingolipids that play a role in metabolic regulation and inflammation. Their elevation has been linked to both beneficial and detrimental outcomes, depending on the context and molecular species [28]. Shabbir and co-workers [29] demonstrated that dietary polyphenols have the capacity to modulate ceramide synthesis and the signaling pathways involved in apoptosis and insulin sensitivity. This suggests the presence of a potential mechanistic link between the intervention and the observed lipidomic shifts.

Collectively, these findings suggest that the daily consumption of a polyphenol-rich beverage can trigger measurable and biologically significant alterations in lipid and metabolic profiles, even under conditions where the intake of polyphenols is otherwise restricted. The increase in distinct lipid species, particularly triglycerides and ceramides, points to targeted metabolic effects rather than global lipid disruption. In contrast to the control group, which exhibited only minor lipid changes, the intervention group demonstrated more substantial and varied alterations, supporting the systemic bioactivity of dietary polyphenols.

### 3.3. Comparison of BSD 3 of Intervention and Control Groups

As we performed a parallel study with one group consuming a placebo without polyphenols and the other one consuming a red fruit juice drink, a comparative analysis of the third BSD revealed a divergent picture when the control group was contrasted with the intervention group, as opposed to when the intervention group’s results were compared at different time points throughout the course of the study (Figure 6).

It is noteworthy that several glycerophospholipids—including ether-linked PEs (PE O-38:5, PE O-36:5, and PE O-34:3), conventional PE (36:4), and LPE (LPE 20:5)—were found to be differentially regulated. Ether-linked PEs (plasmalogens) have been demonstrated to possess antioxidative functions and play a role in membrane integrity and cellular signaling [30,31]. The downregulation observed in the intervention group may be indicative of a compensatory mechanism in response to the increased dietary antioxidants from polyphenols, which could potentially reduce the cellular requirement for endogenous antioxidant lipids, such as plasmalogens.

Furthermore, a notable decrease in FAHFAs (fatty acid esters of hydroxy fatty acids) was observed, specifically in FAHFA 18:0/20:2 and FAHFA 16:0/18:2, in the intervention group. FAHFAs are a class of lipids that have been shown to play a role in the regulation of inflammation and insulin sensitivity [32]. The observed downregulation could be indicative of a modulation of inflammation-related lipid metabolism by polyphenol intake, as polyphenols possess well-documented anti-inflammatory properties [33].

A remarkable finding was the significant downregulation of DG 18:2/18:2, a diacylglycerol that plays a crucial role in signal transduction and serves as an intermediate in lipid metabolism. This phenomenon may be indicative of a decrease in lipolytic activity or an alteration in glycerolipid flux in response to polyphenol exposure. This observation is consistent with that in previous research, which demonstrated that polyphenols can modulate enzymes such as diacylglycerol *O*-acyltransferase (DGAT) and hormone-sensitive lipase [25,34]. This finding also aligns with the results previously published by Rahn et al. in 2023 [20], which demonstrated lower lipase activity in the intervention group than in the control group. However, this effect should not be attributed to the phenol-rich diet, as both groups exhibited a difference in lipase activity at the beginning of the study, which remained unaltered by the intervention.

Conversely, the intervention group exhibited elevated levels of SMs (SM 34:4;O2 and SM 44:5;O2), the triacylglycerol TG 14:0_16:1_16:0, and LPE 20:5. The upregulation of certain sphingolipids may be indicative of altered membrane dynamics or lipid raft composition resulting from the intervention. SMs have been implicated in signaling pathways associated with inflammation and apoptosis, and dietary polyphenols have been documented to exert an influence on sphingolipid metabolism in previous studies. Momchilova et al. (2022) [35] demonstrated that A549 cells treated with resveratrol exhibited a decrease in sphingolipid content within the plasma membrane, while an increase in sphingolipids was observed in the incubation medium. This phenomenon may be attributed to a polyphenol-induced efflux of sphingolipids from the membranes into the medium.

Taken together, these changes suggest that polyphenol intake leads to targeted remodeling of the lipidome, affecting bioactive lipid mediators and membrane components in particular. The direction of the changes—the downregulation of oxidative stress-related lipids (e.g., plasmalogens and FAHFAs) and the upregulation of structural lipids (e.g., SMs)—supports the hypothesis that polyphenol-rich beverages may have beneficial effects by stabilizing the composition of cell membranes and reducing inflammation-related lipid signaling.

## 4. Materials and Methods

### 4.1. Chemicals

The following chemicals were used in LC-MS quality purity: formic acid (Thermo Fisher Scientific, Waltham, MA, USA), ammonium formate (VWR International, Radnor, PA, USA), isopropanol (VWR International, Radnor, PA, USA), methanol (Merck, Darmstadt, Germany), sodium formate (VWR International, Radnor, PA, USA), methyltert-butyl ether (purity 89%, OQEMA, Mönchengladbach, Germany), and triethylamine (purity 99%, Thermo Fisher Scientific, Waltham, MA, USA). Individual lipid standards were all obtained from Avanti^®^ polar Lipids (Alabaster, AL, USA), and they had a purity ≥ 99%: ceramide (18:1/18:1), lysophosphatidyl ethanolamine (16:0/0:0), lysophosphatidylserine (18.1/0:0), phosphatidylethanolamine (18:0/18:1), phosphatidylglycerol (18:0/18:1), phosphatidylinositol (16:/16:0), phosphatidylserine (18:1/18:1), and sphingosine (18:1). LightSPLASH^TM^ LIPIDOMIX^®^ Quantitative Mass and SPLASHTM LIPIDOMIX^®^ Quantitative Mass Spec Internal Standards were obtained from Avanti^®^ polar Lipids, USA. A low-concentration ESI-TOF tuning mix (Agilent Technologies, Waldbronn, Germany) and 20 mM sodium formate buffer were used for calibration.

### 4.2. Human Intervention Study

The study design was previously described in Rahn et al. (2023) [20] and was approved by the Local Ethics Committee of Rhineland-Palatinate, Mainz, Germany (No. 2020-15101). Of the 36 subjects who completed the study, plasma samples were taken from 30 subjects (15 from both the control and intervention groups) to examine the lipid profiles. All volunteers were male, had a BMI of 23.5 ± 1.8 kg m^−2^, and were aged 24.3 ± 2.3 years. The intervention group consumed 750 mL of a polyphenol-rich beverage (PRB) over 8 weeks, and the control group consumed 750 mL of a placebo drink over 8 weeks (the compositions are described in Rahn et al. (2023) [20]). Blood sampling date (BSD) 1 was after one week of washout, BSD 2 was after four weeks of intervention, and BSD 3 was at the end of the study after eight weeks.

### 4.3. Sample Preparation

First, plasma was obtained from the collected human whole blood. For this purpose, blood was collected in 9 mL EDTA monovettes (EDTA: ethylenediaminetetraacetic acid) and centrifuged (10 min, 2500× *g*, room temperature (RT)) so that the solid blood components could be separated from the plasma and settle. The plasma was separated as a yellowish supernatant, aliquoted, and stored at −80 °C.

Sample preparation was carried out manually using liquid–liquid extraction, modified according to a method described by Lerner et al. (2023) [36]. Preparation always took place on ice so that the temperature of the samples could be kept as low as possible to prevent ex vivo variations. The temperature settings of the devices used were also kept as low as possible.

To extract the lipids, 20 µL plasma was added to a 2 mL microreaction vessel and then mixed with 615 µL cold methyl tert-butyl ether (MTBE, 89%, 4 °C), 183 µL cold methanol (MeOH, HPLC grade, 4 °C), and 250 µL cold formic acid (0.1% in bidistilled water (H_2_O_dd_), 4 °C). The samples assigned to the study were additionally spiked with 2 µL isotope-labeled standard (SPLASH^®^ LIPIDOMIX, Avanti), and the MeOH addition was reduced accordingly by this volume. The samples were then vortexed and mixed for a further 10 min in a Thermomixer (Eppendorf, Hamburg, Germany) at 8 °C and 1200 revolutions per minute (rpm). This was followed by a ten-minute centrifugation (13,000 rpm, 4 °C) to enable clean phase separation between the upper organic and lower aqueous phases. Subsequently, 400 µL of the upper organic phase was separated and transferred to a new 1.5 mL microtube. This was followed by evaporation in a nitrogen stream to dryness. The dried extracts were stored at −20 °C until measurement.

Prior to measurement, the extracts were reconstituted by adding 360 µL MeOH (MS grade)/H_2_O_dd_ (9:1, *v*/*v*). The dissolved extracts were placed in an ultrasonic bath for 30 min for better mixing and then centrifuged (10 min, 13,300 rpm, 4 °C) to separate potential solid components such as proteins from the dissolved extracts. Then, 200 µL of the supernatant was filled into vials made of amber glass with an insert and used for measurement.

### 4.4. Untargeted Lipidomics Approach

LC-MS measurements were carried out according to Lerner et al. (2023) [36]. For LC-separation, an Elute UHPLC (Bruker Daltonics, Bremen, Germany) equipped with an Intensity Solo 2 C18 column (100 × 2.1 mm; Bruker Daltonics, Germany) was used. The column oven was kept at 45 °C. In positive mode, 7.5 mM ammonium formate and 0.1% formic acid were added to mobile phase A methanol/H_2_O_dd_ (1/1; *v*/*v*) and mobile phase B methanol/isopropanol (2/8; *v*/*v*). The solvents for negative mode had the same composition as those for positive mode but also contained 0.1% triethylamine. The LC method had a total run time of 20 min and a constant flow rate of 0.2 mL/min. Initially, the mobile phase composition was set to 60% phase A and 40% phase B. After 1 min, the proportion of mobile phase B was increased to 90% by minute 16, followed by a further increase to 99% at minute 16.5 until minute 18. By minute 18.1, the composition was returned to 40% mobile phase B until the end of the run. During the entire analysis, the autosampler was kept at 6 °C. Regarding injection mode, microliter pickup injection was used, with 10 µL in positive mode and 20 µL in negative mode.

MS acquisition was performed in 4D PASEF (Parallel Accumulation Serial Fragmentation) mode using a TimsTOF Pro (Bruker Daltonics, Germany) with electrospray ionization. The MS parameters were as follows: end plate offset (500 V), capillary voltage (4500 V in pos. mode; 3600 V in neg. mode), dry gas (6 L/min), peak detection threshold (100 counts), mass scan range (100–1350 Da), mobility scan range (0.55–1.87 V·s/cm^2^ (pos), 0.55–1.86 V·s/cm^2^ (neg)), and collision energy (35 eV (pos), 45 eV (neg)). Mass calibration was performed at regular intervals using a sodium formate buffer (20 mM). Ion mobility was calibrated using the low-concentration ESI-TOF Tuning Mix from Agilent.

### 4.5. Data Processing

MetaboScape 2021b software from Bruker was used to process the measurement data. Each experiment was processed in positive and negative measurement modes, and the generated bucket tables were then merged.

First, the measurements required for processing were divided according to their affiliation with the study group and blood sampling. This created two “sample groups” with the titles “Group” and “BE” for the blood sampling date. The sample group “Group” had the attributes “Intervention” and “Control” for the assignment of the test subject’s measured blood sample to the intervention or control group. In addition, the respective blood sampling dates, i.e., “1st BE,” “2nd BE,” and “3rd BE,” were assigned to “BE”. The QC samples were marked as QC by the program and given the name “Quality Control” in both sample groups. Due to the strictly identical preparation of all samples and the generally low individual variation in plasma lipids in human blood samples, the filter parameters “Minimum # Features for Extraction” and “Presence of features in minimum # of analyses” were set to 80% of the total number of analyses processed. Filtering of the presence of features within the respective group was not carried out. In addition, the intensity threshold was set to 100, and the minimum 4D peak size was set to 75 in the “T-Rex Method Configuration”. The seed ion [M+AcO]^+^ was also added to the ion deconvolution parameterization, with the intention of detecting as many isotope-labeled lipids of the internal standard as possible. Following the processing of the experiments in the respective polarity, the generated bucket tables were merged to evaluate the entire experiment. The retention time tolerance between the tables was set to 25 s, and the mass tolerance was set to 2 ppm. After merging the bucket tables, annotations were first made using the spectral libraries (Bruker) and rule-based lipid annotation. A manually created annotation list of the isotope-labeled lipids contained in the internal standard was also used. To normalize the data, the isotope-labeled lipid PG 15:0_18:1(d7) of the internal mixed standard was used in each case, as it was detected and annotated in all processing steps without exception.

### 4.6. Software

The following software was used: Bruker Compass HyStar 5.1 Version: 5.1.8.1; Bruker Compass DataAnalysis Version 6.0; Compass 2023 for timsTOF Series otofControl Version 6.2; and Bruker Compass MetaboScape^®^ 2021b Version 7.0.1 (Bruker Daltonik GmbH, Bremen, Deutschland). The R package Tidy Verse [37] was used to visualize the data.

## 5. Conclusions

In summary, the present study demonstrates that dietary polyphenol intake exerts a substantial influence on the human plasma lipidome, even under conditions of strict dietary control. In the control group, minor yet statistically significant temporal shifts in specific lipid classes—particularly PEs and ether-linked lipids—demonstrated the dynamic nature of lipid metabolism and underscored the importance of time-matched controls in longitudinal lipidomics studies. Conversely, the intervention group, which consumed a polyphenol-rich beverage, exhibited significant lipidomic remodeling. This included elevations in select TGs and SLs, alongside reductions in oxidative stress-associated lipids, such as plasmalogens and FAHFAs. A comparative analysis further confirmed that polyphenol intake modulated distinct lipid pathways, including those related to membrane integrity, inflammation, and lipid signaling.

Taken together, our findings provide robust evidence that dietary polyphenols exert systemic bioactive effects on lipid metabolism, reinforcing their potential as modulators of metabolic health. These results underscore the importance of incorporating lipidomics into nutritional intervention studies to elucidate subtle yet biologically significant metabolic adaptations.

## Data Availability

The data are available upon request.

## Abbreviations

The following abbreviations are used in this manuscript:

AMPK	AMP-activated protein kinase
BE	Blood sampling date
BMI	Body mass index
CE	Cholesteryl ester
Cer	Ceramide
DAG	Diacylglycerol
DG	Diacylglyceride
DGAT	Diacylglycerol O-acyltransferase
EDTA	Ethylenediaminetetraacetic acid
FAHFA	Fatty acid ester of hydroxy fatty acid
GAE	Gallic acid equivalent
HDL	High-density lipoprotein
LC-MS	Liquid chromatography–mass spectrometry
LPC	Lysophosphatidylcholine
LPE	Lysophosphatidylethanolamine
MeOH	Methanol
MTBE	Methyl tert-butyl ether
PC	Phosphatidylcholine
PE	Phosphatidylethanolamine
PI	Phosphatidylinositol
PL	Phospholipid
PRB	Polyphenol-rich beverage
QC	Quality control
RT	Room temperature
SL	Sphingolipid
SM	Sphingomyelin
ST	Sulfatide
TG	Triacylglyceride (triglyceride)
